# Progress in Understanding and Enhancing Rice Tolerance to Biotic and Abiotic Stresses

**DOI:** 10.3390/plants13223206

**Published:** 2024-11-15

**Authors:** Weixun Wu, Yingxin Zhang, Guiai Jiao, Xiangjin Wei

**Affiliations:** State Key Laboratory of Rice Biology and Breeding, China National Center for Rice Improvement, China National Rice Research Institute, Hangzhou 311400, China; wuweixun@caas.cn (W.W.); zhangyingxin@caas.cn (Y.Z.); jiaoguiai@caas.cn (G.J.)

Rice growth and development occur in several distinct stages: a seedling stage, a vegetative stage, a reproductive stage, and maturity. Throughout these stages, rice is frequently subjected to various biotic stresses, such as diseases, pests, and weeds, as well as abiotic stresses, including extreme temperatures, drought, waterlogging, salinity, heavy metals, and nutrient deficiencies. These stressors negatively impact rice yield and quality to varying degrees, resulting in significant annual economic losses for the global rice industry. Consequently, developing rice varieties with enhanced adaptability remains necessary.

Among biotic stresses, disease is a primary threat in rice cultivation, with rice blast, sheath blight, and rice false smut numbering among the most detrimental. The main strategies for disease control include using disease-resistant cultivars and chemical interventions. In resistance breeding, combining multiple resistance genes can widen disease resistance and improve overall crop resilience. Peng et al. [1] successfully introduced three resistance genes, *Pigm*, *Pi48*, and *Pi49*, into the photothermosensitive male-sterile line Chuang5S using marker-assisted selection (MAS) and the RICE10K SNP chip. This approach allowed the rapid breeding of an enhanced line that retained a genetic profile similar to that of Chuang5S. The resulting rice varieties demonstrated improved blast resistance without compromised yields or agronomic traits, indicating the promising potential of using gene stacking to provide broad-spectrum disease resistance in rice breeding.

Lesion-mimic mutants (LMMs) in rice, often associated with hypersensitive response (HR), are significantly influenced by environmental factors such as temperature, light, and humidity. Certain chloroplast maculoid mutants activate defense responses, augmenting disease resistance. Liu et al. characterized the rice spot-like mutant *spl42*, which is associated with *SPOTTED LEAF42* (*SPL42*). This gene encodes porphobilinogen deaminase (PBGD), an enzyme crucial in the porphyrin biosynthesis pathway during chlorophyll synthesis. Mutations in *SPL42* reduced PBGD activity, altering the expression of several genes involved in chlorophyll biosynthesis and defense responses. The *spl42* mutant displayed red-brown spotted leaves, reduced chlorophyll and carotenoid contents, and an abnormal leaf ultrastructure [2]. These findings explain *SPL42′*s role in chlorophyll synthesis and leaf development in rice, providing insights helpful for enhancing rice yield and quality.

Weeds in paddy fields constitute a major biological stressor for rice cultivation, and waterlogging treatment is a common strategy for weed management. Developing flood-resistant rice varieties is therefore essential for effective weed management and can decrease reliance on pesticides. Kostylev et al. [3] successfully incorporated the *Sub1A* gene for long-term waterlogging tolerance into economically viable rice varieties, using anther culture to produce dihaploids resilient to extended waterlogging. Evaluation of these samples revealed that certain samples exhibited rapid expansion of the first leaf and enhanced nutrient accumulation under flooded conditions, indicating enhanced flood resistance. These results contribute valuable genetic resources for rice breeding, helping to produce varieties with improved flood tolerance.

Rice is also significantly impacted by abiotic stressors, particularly extreme temperatures, waterlogging, and high salinity. The optimal temperatures for rice vary across growth stages, and deviations from these optimal temperatures can severely affect rice development. The maximum temperature during the rice-filling period should not exceed 35 °C. Elevated temperatures during this stage accelerate the accumulation of stored substances in seeds, resulting in grain-filling defects and reductions in yield and quality. Li et al. [4] studied the effects of high temperatures on seed development and successfully cloned *OsLEA1b*, a gene that responds to high temperatures. Expressed in rice endosperm, *OsLEA1b* encodes a protein involved in the heat stress response during grain filling. The *oslea1b* mutant exhibited an abnormal starch grain structure, a white endosperm phenotype, and significant reductions in grain weight and the number of grains per panicle. The starch composition also changed in the mutant, with fewer short-chain starches and more long-chain starches, especially under high temperatures. This study demonstrates *OsLEA1b*’s regulatory role in starch synthesis under high-temperature conditions, impacting rice quality and yield.

Mesocotyl elongation, crucial for waterlogging resilience during the seedling stage, helps establish deep sowing tolerance and enhances drought resilience in directly seeded rice. Varieties with longer mesocotyls can better break the soil, reducing anaerobic stress and increasing emergence and survival rates. Feng et al. [5] studied mesocotyl length in the Rice Diversity Panel 1 (RDP1) and Hanyou 73 (HY73) recombinant inbred lines (RILs) under dark germination conditions. They then employed genome-wide association studies (GWAS) of RDP1 and linkage mapping of HY73 RIL to identify genetic loci associated with mesocotyl elongation. Dynamic RNA sequencing was used to further validate the candidate genes, integrating phenotypic and genomic data to inform future rice breeding. This research provides insights into genetic factors influencing mesocotyl elongation, offering guidance for developing rice varieties with enhanced adaptability.

Soil salinization poses a significant challenge regarding rice yield and quality, necessitating insights into molecular mechanisms that enhance salt tolerance for breeding purposes. The *CIPK* gene family has been shown to play diverse roles in modulating salt tolerance, with *OsCIPK24* being linked specifically to this trait. Recent findings made by Zhou et al. [6] demonstrated that *OsCIPK9* knockout mutants exhibited improved salt tolerance, while overexpressing *OsCIPK9* resulted in heightened salt sensitivity. This study also found that *OsCIPK9* interacts with *OsSOS3*, suggesting the former’s regulator role in salt-related transporters. RNA-seq data showed a pronounced *OsCIPK9* response to salt stress, likely through downstream salt tolerance genes. These findings demonstrate that *OsCIPK9* functions as a negative regulator of salt tolerance, offering insights for further research on salt tolerance mechanisms in rice. CRISPR technology could expedite assessments of other *OsCIPK* family genes, contributing to the development of a clearer picture of the regulatory pathway for rice salt tolerance.

YT521-B homology (YTH) domain proteins are essential for rice growth and environmental stressor responses, particularly in RNA modifications like N6-methyladenosine (m6A), which are crucial for gene regulation. In a recent study, Ma et al. [7] used CRISPR-Cas9 to generate YTH-deficient rice mutants, which displayed reduced plant heights, fewer spikelets per panicle, and lower setting rates and grain weights, indicating YTH protein’s importance in rice growth and development. Additionally, certain YTH protein mutants exhibited variable tolerance to saline–alkali stress, demonstrating the YTH protein’s role in stress response.

The cyclic nucleotide-gated channel (CNGC) family significantly modulates plant immunity and abiotic stress responses. Wang et al. [8] identified 16 *CNGC* genes in rice, examining their chromosomal locations, physicochemical properties, and subcellular localization. By analyzing haplotype (gcHap) diversity across 3010 genomes, the researchers explored genetic variation and selection pressures acting on *CNGC* genes in rice populations. Their study reveals the advantageous alleles of *OsCNCGs* that may enhance rice yield and abiotic stress tolerance, marking these alleles as key resources for future rice breeding.

Heading date is an important agronomic trait that affects rice maturity, adaptability, and yield. Identifying genes associated with heading date can improve rice’s regional and seasonal adaptability. Despite numerous quantitative trait loci (QTLs) linked to heading date, few have been successfully cloned. Sohail et al. [9] used 76 chromosome segment substitution lines (CSSLs) derived from Zhonghui9308 (ZH9308) and XieqingzaoB (XQZB) to identify 14 QTLs associated with heading date, including a fine-mapped *qHD7b* on chromosome 7. Their results showed that the *qHD7b*^XQZB^ allele was non-functional at the *Ghd7* locus, while the *qHD7b*^ZH9308^ allele was functional. The functional *Ghd7* allele can extend the heading date in early-maturing varieties, potentially enhancing yield. Conversely, the non-functional *Ghd7* allele can accelerate maturation in late-maturing varieties. Additionally, several rice heading date genes also regulate abiotic stress responses, such as salinity and alkalinity tolerance.

The substantial advancements made over the past decade have broadened our understanding of rice responses to biotic and abiotic stresses. Breeding resistant varieties remains an effective solution to these challenges. Using well-characterized germplasm resources and marker-assisted selection (MAS) to introduce resistance genes has been effective in breeding programs. Consequently, the investigation of genes and haplotypes that have adapted to diverse environments provides valuable genetic resources for breeding additional resistant varieties. Recently, gene-editing and high-throughput sequencing technologies—such as Kompetitive Allele Specific PCR (KASP), whole-genome sequencing, targeted sequencing, and single-nucleotide polymorphism (SNP) breeding chips—have been widely employed, accelerating the development of resistant varieties and reducing breeding timelines.

This Special Issue aims to provide a comprehensive overview of advancements in the resistance of rice to biotic and abiotic stresses, covering recent research on molecular breeding and germplasm improvement. We believe that the articles and insights discussed here will provide readers with relevant knowledge of resistance resources with which to sustainably increase crop yields—a necessity for ensuring future food security. Combining modern biotechnology with traditional breeding methods, these studies offer actionable strategies for addressing stress-related challenges in rice production and supporting sustainable agriculture.
